# *catena*-Poly[[[tetra­aqua­manganese(II)]-μ-4,4′-bipyridine] bis­(3-hydroxy­cinnamate) dihydrate]

**DOI:** 10.1107/S1600536809028360

**Published:** 2009-07-22

**Authors:** Zhi-Wei Tang, Jun-Dan Fu, Long-Ping Jiang, Yi-Hang Wen

**Affiliations:** a Zhejiang Key Laboratory for Reactive Chemistry on Solid Surfaces, Institute of Physical Chemistry, Zhejiang Normal University, Jinhua, Zhejiang 321004, People’s Republic of China

## Abstract

The title compound, {[Mn(C_10_H_8_N_2_)(H_2_O)_4_](C_9_H_7_O_3_)_2_·2H_2_O}_*n*_, was obtained by the hydro­thermal reaction of manganese chloride with mixed 3-hydroxy­lcinnamic acid (H_2_ *L*) and 4,4′-bipyridine (4,4′-bipy) ligands. The structure contains [Mn(C_10_H_8_N_2_)(H_2_O)_4_]^2+^ cations with the Mn^II^ atoms lying on a centres of inversion and bridged into a linear chain along the *a* axis by 4,4′-bipy ligands, surrounded by H*L* ^−^ anions and uncoordinated water mol­ecules. Extensive O—H⋯O hydrogen-bonding and weak π–π inter­actions [centroid–centroid distance = 3.7572  (3) Å] between the constituents lead to the formation of a three-dimensional supra­molecular network.

## Related literature

For potential applications of compounds with supramolecular architectures, see: Niu *et al.* (2008 ▶); Xue *et al.* (2007 ▶); Ye *et al.* (2005 ▶); Zhang *et al.* (2009 ▶). For the synthesis of supra­molecular coordination compounds containing 4-pyridyl and carboxyl­ate groups, see: Feng *et al.* (2008 ▶); He *et al.* (2007 ▶); Li *et al.* (2008 ▶).
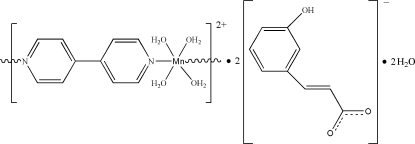


## Experimental

### 

#### Crystal data

[Mn(C_10_H_8_N_2_)(H_2_O)_4_](C_9_H_7_O_3_)_2_·2H_2_O
*M* *_r_* = 645.51
Monoclinic, 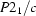

*a* = 11.6620 (12) Å
*b* = 11.2726 (13) Å
*c* = 11.6238 (13) Å
β = 96.520 (9)°
*V* = 1518.2 (3) Å^3^
*Z* = 2
Mo *K*α radiation
μ = 0.50 mm^−1^
*T* = 296 K
0.21 × 0.14 × 0.07 mm

#### Data collection

Bruker APEXII area-detector diffractometer
Absorption correction: multi-scan (*SADABS*; Sheldrick, 1996 ▶) *T*_min_ = 0.92, *T*_max_ = 0.97
13208 measured reflections
3513 independent reflections
2293 reflections with *I* > 2σ(*I*)
*R*_int_ = 0.060

#### Refinement

*R*[*F* ^2^ > 2σ(*F* ^2^)] = 0.044
*wR*(*F* ^2^) = 0.118
*S* = 1.04
3513 reflections
217 parameters
10 restraints
H atoms treated by a mixture of independent and constrained refinement
Δρ_max_ = 0.21 e Å^−3^
Δρ_min_ = −0.31 e Å^−3^

### 

Data collection: *APEX2* (Bruker, 2006 ▶); cell refinement: *SAINT* (Bruker, 2006 ▶); data reduction: *SAINT*; program(s) used to solve structure: *SHELXS97* (Sheldrick, 2008 ▶); program(s) used to refine structure: *SHELXL97* (Sheldrick, 2008 ▶); molecular graphics: *SHELXTL* (Sheldrick, 2008 ▶); software used to prepare material for publication: *SHELXTL*.

## Supplementary Material

Crystal structure: contains datablocks I, global. DOI: 10.1107/S1600536809028360/at2846sup1.cif

Structure factors: contains datablocks I. DOI: 10.1107/S1600536809028360/at2846Isup2.hkl

Additional supplementary materials:  crystallographic information; 3D view; checkCIF report

## Figures and Tables

**Table 1:** Hydrogen-bond geometry (Å, °)

*D*—H⋯*A*	*D*—H	H⋯*A*	*D*⋯*A*	*D*—H⋯*A*
O3*W*—H3*WA*⋯O2^i^	0.832 (16)	1.912 (18)	2.738 (2)	171 (3)
O1*W*—H1*WA*⋯O1^ii^	0.833 (17)	1.888 (17)	2.719 (2)	174 (3)
O2*W*—H2*WA*⋯O3*W*^iii^	0.815 (17)	2.024 (17)	2.838 (3)	176 (3)
O3*W*—H3*WB*⋯O2^iv^	0.842 (16)	1.902 (18)	2.741 (2)	174 (3)
O2*W*—H2*WB*⋯O1^v^	0.832 (16)	1.878 (16)	2.702 (2)	171 (3)

Symmetry codes: (i) 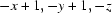; (ii) 
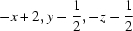
; (iii) 
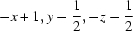
; (iv) 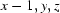; (v) 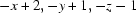.

**Fig. 1.**
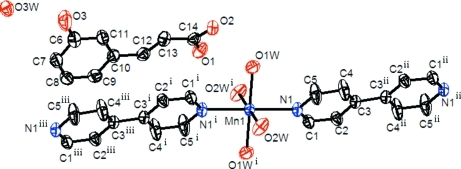
The molecular structure of the title compound, showing the atom-numbering scheme. Displacement ellipsoids are drawn at the 30% probability level and H atoms have been omitted for clarity. [Symmetry codes: (i) 2 - x, 1 - y, -1 - z; (ii) 3 - x, 1 - y, 1 - z; (iii) -1 + x, y, z]

**Fig. 2.**
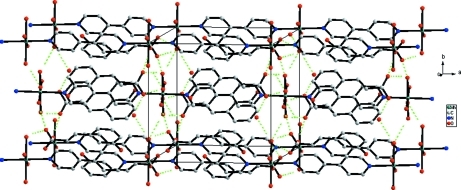
Packing diagram showing hydrogen bonds as dashed lines. All H atoms have been omitted for clarity.

## supplementary crystallographic information

### Comment

The construction of supramolecular architectures based on metal and organic building blocks is currently of great interest for their aesthetic architectures and potential functions such as adsorption, ion exchange, magnetic and luminescent materials (Niu *et al.*, 2008; Xue *et al.*, 2007; Ye *et al.*, 2005; Zhang *et al.*, 2009). Recently, we are interested in the synthesis of novel supramolecular coordination compounds which contain not only 4-pyridyl but also carboxylate groups in the crystal structure (He *et al.*, 2007; Feng *et al.*, 2008; Li *et al.*, 2008). Here we report the crystal structure of the title compound, [Mn(C_10_H_8_N_2_)(H_2_O)_4_]_2_.2(C_9_H_7_O_3_).2H_2_O, (I).

The present X-ray single-crystal diffraction study reveals that (I) is a new coordination polymer involving Mn^2+^ and 3-hydroxycinnamate anions, as shown in Fig. 1. The Mn^II^ is hexacoordinated in an octahedral manner by four water molecules in the equatorial plane and two N atoms in the axial positions from two 4,4'-bipyridine molecules. The bond lengths of Mn—N and Mn—O are 2.2863 (17) Å and in the range 2.1641 (15)—2.1675 (17) Å, respectively. As shown in Fig. 2, the linear cationic chains, 3-Hydroxycinnamate anions and lattice water molecules are linked together through a series of O—H···O bonds with the hydrogen bonds lengths in the range of 2.702 (2)—2.838 (3) Å and bond angles between 171 (3) and 176 (3) °. The extensive hydrogen bonds together with the weak π-π interactions between hca^-^ anions and 4,4'-bipyridine (the centroid-to-centroid distance is 3.7572 Å) stabilize the crystal structure, forming a three-dimensional network.

### Experimental

MnCl_2_.4H_2_O (0.0973 g, 0.5 mmol), 3-hydroxycinnamic acid (0.1619 g, 1 mmol), NaOH (0.0405 g, 1 mmol), 4,4'-bipy (0.1562 g, 1 mmol) and H_2_O-ethanol (4:1, 15 mL) was sealed in a 25 ml stainless-steel reactor with a Telflon liner and was heated at 433 K for 3 d, then the reactor was cooled slowly to room temperature. The solution was filtered, giving yellow single crystals suitable for X-ray analysis in yield 30%.

### Refinement

The carbon-bound H-atoms were positioned geometrically and included in the refinement using a riding model [C—H 0.93 Å *U*_iso_(H) = 1.2*U*_eq_(C)]. The water and hydroxyl H atoms were located from different maps, and their positions were refined isotropically, with O—H distances fixed by O_water_—H = 0.85 (2) Å, O_hydroxyl_—H = 0.96 (2) Å and H—H = 1.30 (2) Å, their displacement parameters were set to 1.5*U*_eq_(O_water_) and 1.2*U*_eq_(O_hydroxyl_).

### Crystal data

**Table:**

[Mn(C_10_H_8_N_2_)(H_2_O)_4_](C_9_H_7_O_3_)_2_·2H_2_O	*F*(000) = 674
*M**_r_* = 645.51	*D*_x_ = 1.412 Mg m^−^^3^
Monoclinic, *P*2_1_/*c*	Mo *K*α radiation, λ = 0.71073 Å
Hall symbol: -P 2ybc	Cell parameters from 1488 reflections
*a* = 11.6620 (12) Å	θ = 1.8–27.7°
*b* = 11.2726 (13) Å	µ = 0.50 mm^−^^1^
*c* = 11.6238 (13) Å	*T* = 296 K
β = 96.520 (9)°	Block, yellow
*V* = 1518.2 (3) Å^3^	0.21 × 0.14 × 0.07 mm
*Z* = 2	

### Data collection

**Table:**

Bruker APEXII area-detector diffractometer	3513 independent reflections
Radiation source: fine-focus sealed tube	2293 reflections with *I* > 2σ(*I*)
graphite	*R*_int_ = 0.060
ω scans	θ_max_ = 27.7°, θ_min_ = 1.8°
Absorption correction: multi-scan (*SADABS*; Sheldrick, 1996)	*h* = −15→14
*T*_min_ = 0.92, *T*_max_ = 0.97	*k* = −14→14
13208 measured reflections	*l* = −15→15

### Refinement

**Table:**

Refinement on *F*^2^	Primary atom site location: structure-invariant direct methods
Least-squares matrix: full	Secondary atom site location: difference Fourier map
*R*[*F*^2^ > 2σ(*F*^2^)] = 0.044	Hydrogen site location: inferred from neighbouring sites
*wR*(*F*^2^) = 0.118	H atoms treated by a mixture of independent and constrained refinement
*S* = 1.04	*w* = 1/[σ^2^(*F*_o_^2^) + (0.0535*P*)^2^ + 0.0147*P*] where *P* = (*F*_o_^2^ + 2*F*_c_^2^)/3
3513 reflections	(Δ/σ)_max_ < 0.001
217 parameters	Δρ_max_ = 0.21 e Å^−^^3^
10 restraints	Δρ_min_ = −0.31 e Å^−^^3^

### Special details

**Table:**

Geometry. All e.s.d.'s (except the e.s.d. in the dihedral angle between two l.s. planes) are estimated using the full covariance matrix. The cell e.s.d.'s are taken into account individually in the estimation of e.s.d.'s in distances, angles and torsion angles; correlations between e.s.d.'s in cell parameters are only used when they are defined by crystal symmetry. An approximate (isotropic) treatment of cell e.s.d.'s is used for estimating e.s.d.'s involving l.s. planes.
Refinement. Refinement of *F*^2^ against ALL reflections. The weighted *R*-factor *wR* and goodness of fit *S* are based on *F*^2^, conventional *R*-factors *R* are based on *F*, with *F* set to zero for negative *F*^2^. The threshold expression of *F*^2^ > σ(*F*^2^) is used only for calculating *R*-factors(gt) *etc*. and is not relevant to the choice of reflections for refinement. *R*-factors based on *F*^2^ are statistically about twice as large as those based on *F*, and *R*- factors based on ALL data will be even larger.

### Fractional atomic coordinates and isotropic or equivalent isotropic displacement parameters (Å^2^)

**Table:**

	*x*	*y*	*z*	*U*_iso_*/*U*_eq_	
Mn1	1.0000	0.5000	−0.5000	0.02932 (15)	
C1	1.24961 (19)	0.5744 (2)	−0.5720 (2)	0.0384 (6)	
H1A	1.2060	0.6305	−0.6164	0.046*	
C2	1.36718 (19)	0.5795 (2)	−0.5695 (2)	0.0383 (6)	
H2A	1.4008	0.6376	−0.6116	0.046*	
C3	1.43615 (18)	0.4985 (2)	−0.50437 (18)	0.0316 (5)	
C4	1.3781 (2)	0.4144 (3)	−0.4478 (3)	0.0657 (9)	
H4A	1.4193	0.3561	−0.4042	0.079*	
C5	1.2599 (2)	0.4158 (3)	−0.4553 (3)	0.0652 (9)	
H5A	1.2239	0.3575	−0.4156	0.078*	
C6	0.3636 (2)	0.6015 (2)	−0.1614 (2)	0.0479 (6)	
C7	0.2912 (2)	0.6654 (3)	−0.2419 (2)	0.0529 (7)	
H7A	0.2115	0.6598	−0.2423	0.063*	
C8	0.3368 (2)	0.7364 (3)	−0.3205 (2)	0.0519 (7)	
H8A	0.2881	0.7802	−0.3734	0.062*	
C9	0.4546 (2)	0.7436 (2)	−0.3218 (2)	0.0479 (7)	
H9A	0.4849	0.7928	−0.3751	0.057*	
C10	0.5288 (2)	0.6779 (2)	−0.2439 (2)	0.0397 (6)	
C11	0.4815 (2)	0.6072 (2)	−0.1637 (2)	0.0454 (6)	
H11A	0.5299	0.5630	−0.1108	0.055*	
C12	0.6538 (2)	0.6860 (2)	−0.2478 (2)	0.0403 (6)	
H12A	0.6795	0.7441	−0.2956	0.048*	
C13	0.7334 (2)	0.6189 (2)	−0.1900 (2)	0.0425 (6)	
H13A	0.7098	0.5621	−0.1397	0.051*	
C14	0.8572 (2)	0.6292 (2)	−0.20085 (19)	0.0377 (6)	
N1	1.19366 (15)	0.49461 (16)	−0.51532 (16)	0.0353 (4)	
O1	0.89468 (15)	0.71680 (16)	−0.25223 (15)	0.0487 (5)	
O1W	1.02880 (17)	0.43379 (17)	−0.32434 (13)	0.0502 (5)	
H1WA	1.049 (3)	0.3672 (17)	−0.298 (3)	0.075*	
H1WB	1.004 (3)	0.472 (2)	−0.272 (2)	0.075*	
O2	0.92237 (14)	0.54517 (16)	−0.15829 (14)	0.0443 (4)	
O2W	0.98772 (18)	0.31913 (16)	−0.56349 (17)	0.0531 (5)	
H2WA	0.955 (3)	0.260 (2)	−0.542 (3)	0.080*	
H2WB	1.022 (3)	0.300 (3)	−0.620 (2)	0.080*	
O3	0.32335 (17)	0.5320 (2)	−0.0786 (2)	0.0769 (7)	
H3	0.2485 (18)	0.551 (3)	−0.074 (3)	0.092*	
O3W	0.11487 (15)	0.61016 (17)	−0.01468 (16)	0.0480 (5)	
H3WA	0.110 (2)	0.566 (2)	0.0420 (18)	0.072*	
H3WB	0.058 (2)	0.593 (3)	−0.0630 (19)	0.072*	

### Atomic displacement parameters (Å^2^)

**Table:**

	*U*^11^	*U*^22^	*U*^33^	*U*^12^	*U*^13^	*U*^23^
Mn1	0.0232 (3)	0.0318 (3)	0.0335 (2)	0.0007 (2)	0.00570 (18)	0.0012 (2)
C1	0.0256 (13)	0.0441 (15)	0.0449 (13)	0.0012 (10)	0.0012 (10)	0.0104 (11)
C2	0.0261 (13)	0.0458 (15)	0.0431 (12)	−0.0034 (10)	0.0039 (10)	0.0140 (11)
C3	0.0237 (11)	0.0340 (12)	0.0377 (11)	0.0004 (10)	0.0063 (9)	0.0002 (10)
C4	0.0266 (15)	0.066 (2)	0.106 (2)	0.0109 (13)	0.0130 (14)	0.0522 (18)
C5	0.0276 (15)	0.064 (2)	0.106 (2)	0.0062 (13)	0.0183 (15)	0.0493 (18)
C6	0.0354 (15)	0.0512 (17)	0.0569 (15)	0.0013 (12)	0.0047 (12)	−0.0016 (13)
C7	0.0354 (15)	0.066 (2)	0.0553 (16)	0.0066 (13)	−0.0035 (12)	−0.0148 (14)
C8	0.0446 (17)	0.0655 (19)	0.0433 (14)	0.0151 (14)	−0.0059 (12)	−0.0087 (13)
C9	0.0500 (18)	0.0548 (17)	0.0384 (13)	0.0082 (13)	0.0030 (12)	−0.0054 (12)
C10	0.0356 (14)	0.0437 (15)	0.0393 (12)	0.0037 (11)	0.0018 (10)	−0.0100 (11)
C11	0.0318 (14)	0.0513 (16)	0.0526 (15)	0.0061 (12)	0.0019 (11)	0.0024 (12)
C12	0.0404 (15)	0.0417 (15)	0.0396 (12)	0.0002 (12)	0.0080 (11)	−0.0057 (11)
C13	0.0372 (14)	0.0433 (15)	0.0485 (14)	−0.0013 (11)	0.0106 (11)	0.0009 (12)
C14	0.0373 (14)	0.0422 (14)	0.0347 (12)	−0.0033 (11)	0.0082 (10)	−0.0094 (11)
N1	0.0240 (10)	0.0362 (11)	0.0467 (10)	0.0030 (9)	0.0089 (8)	0.0047 (9)
O1	0.0520 (11)	0.0422 (11)	0.0552 (10)	−0.0082 (9)	0.0199 (9)	−0.0059 (8)
O1W	0.0615 (13)	0.0542 (12)	0.0363 (9)	0.0249 (10)	0.0118 (8)	0.0075 (8)
O2	0.0343 (10)	0.0513 (11)	0.0481 (9)	0.0056 (8)	0.0079 (8)	−0.0013 (8)
O2W	0.0644 (13)	0.0367 (10)	0.0639 (12)	−0.0111 (9)	0.0321 (10)	−0.0091 (9)
O3	0.0394 (12)	0.0962 (17)	0.0977 (16)	0.0068 (12)	0.0190 (12)	0.0337 (14)
O3W	0.0375 (11)	0.0509 (12)	0.0554 (11)	−0.0055 (9)	0.0040 (8)	0.0011 (9)

### Geometric parameters (Å, °)

**Table:**

Mn1—O1W	2.1641 (15)	C7—H7A	0.9300
Mn1—O1W^i^	2.1641 (15)	C8—C9	1.378 (4)
Mn1—O2W^i^	2.1675 (17)	C8—H8A	0.9300
Mn1—O2W	2.1675 (17)	C9—C10	1.393 (3)
Mn1—N1^i^	2.2863 (17)	C9—H9A	0.9300
Mn1—N1	2.2863 (17)	C10—C11	1.388 (3)
C1—N1	1.329 (3)	C10—C12	1.466 (3)
C1—C2	1.369 (3)	C11—H11A	0.9300
C1—H1A	0.9300	C12—C13	1.320 (3)
C2—C3	1.385 (3)	C12—H12A	0.9300
C2—H2A	0.9300	C13—C14	1.468 (3)
C3—C4	1.375 (3)	C13—H13A	0.9300
C3—C3^ii^	1.482 (4)	C14—O1	1.258 (3)
C4—C5	1.371 (3)	C14—O2	1.278 (3)
C4—H4A	0.9300	O1W—H1WA	0.833 (17)
C5—N1	1.323 (3)	O1W—H1WB	0.825 (16)
C5—H5A	0.9300	O2W—H2WA	0.815 (17)
C6—O3	1.364 (3)	O2W—H2WB	0.832 (16)
C6—C11	1.380 (3)	O3—H3	0.908 (18)
C6—C7	1.388 (4)	O3W—H3WA	0.832 (16)
C7—C8	1.368 (4)	O3W—H3WB	0.842 (16)
			
O1W—Mn1—O1W^i^	180.00 (10)	C8—C7—H7A	120.0
O1W—Mn1—O2W^i^	90.34 (8)	C6—C7—H7A	120.0
O1W^i^—Mn1—O2W^i^	89.66 (8)	C7—C8—C9	120.4 (3)
O1W—Mn1—O2W	89.66 (8)	C7—C8—H8A	119.8
O1W^i^—Mn1—O2W	90.34 (8)	C9—C8—H8A	119.8
O2W^i^—Mn1—O2W	180.00 (10)	C8—C9—C10	120.6 (3)
O1W—Mn1—N1^i^	89.11 (7)	C8—C9—H9A	119.7
O1W^i^—Mn1—N1^i^	90.89 (7)	C10—C9—H9A	119.7
O2W^i^—Mn1—N1^i^	88.63 (7)	C11—C10—C9	118.5 (2)
O2W—Mn1—N1^i^	91.37 (7)	C11—C10—C12	121.9 (2)
O1W—Mn1—N1	90.89 (7)	C9—C10—C12	119.6 (2)
O1W^i^—Mn1—N1	89.11 (7)	C6—C11—C10	120.8 (2)
O2W^i^—Mn1—N1	91.37 (7)	C6—C11—H11A	119.6
O2W—Mn1—N1	88.63 (7)	C10—C11—H11A	119.6
N1^i^—Mn1—N1	180.0	C13—C12—C10	126.4 (2)
N1—C1—C2	124.4 (2)	C13—C12—H12A	116.8
N1—C1—H1A	117.8	C10—C12—H12A	116.8
C2—C1—H1A	117.8	C12—C13—C14	123.6 (2)
C1—C2—C3	120.1 (2)	C12—C13—H13A	118.2
C1—C2—H2A	119.9	C14—C13—H13A	118.2
C3—C2—H2A	119.9	O1—C14—O2	122.8 (2)
C4—C3—C2	115.4 (2)	O1—C14—C13	120.0 (2)
C4—C3—C3^ii^	122.0 (3)	O2—C14—C13	117.1 (2)
C2—C3—C3^ii^	122.6 (2)	C5—N1—C1	115.2 (2)
C5—C4—C3	120.5 (2)	C5—N1—Mn1	119.98 (15)
C5—C4—H4A	119.7	C1—N1—Mn1	124.44 (15)
C3—C4—H4A	119.7	Mn1—O1W—H1WA	132 (2)
N1—C5—C4	124.3 (2)	Mn1—O1W—H1WB	119 (2)
N1—C5—H5A	117.8	H1WA—O1W—H1WB	108 (2)
C4—C5—H5A	117.8	Mn1—O2W—H2WA	133 (2)
O3—C6—C11	117.6 (2)	Mn1—O2W—H2WB	119 (2)
O3—C6—C7	122.8 (2)	H2WA—O2W—H2WB	108 (2)
C11—C6—C7	119.6 (3)	C6—O3—H3	108 (2)
C8—C7—C6	120.1 (3)	H3WA—O3W—H3WB	105 (2)
			
N1—C1—C2—C3	−0.3 (4)	C9—C10—C12—C13	−171.1 (2)
C1—C2—C3—C4	1.7 (4)	C10—C12—C13—C14	177.8 (2)
C1—C2—C3—C3^ii^	−178.3 (3)	C12—C13—C14—O1	11.9 (4)
C2—C3—C4—C5	−1.6 (4)	C12—C13—C14—O2	−166.8 (2)
C3^ii^—C3—C4—C5	178.3 (3)	C4—C5—N1—C1	1.2 (4)
C3—C4—C5—N1	0.2 (5)	C4—C5—N1—Mn1	−171.9 (3)
O3—C6—C7—C8	178.0 (3)	C2—C1—N1—C5	−1.2 (4)
C11—C6—C7—C8	−2.1 (4)	C2—C1—N1—Mn1	171.64 (19)
C6—C7—C8—C9	1.1 (4)	O1W—Mn1—N1—C5	29.3 (2)
C7—C8—C9—C10	0.6 (4)	O1W^i^—Mn1—N1—C5	−150.7 (2)
C8—C9—C10—C11	−1.4 (3)	O2W^i^—Mn1—N1—C5	119.6 (2)
C8—C9—C10—C12	179.3 (2)	O2W—Mn1—N1—C5	−60.4 (2)
O3—C6—C11—C10	−178.7 (2)	O1W—Mn1—N1—C1	−143.18 (19)
C7—C6—C11—C10	1.4 (4)	O1W^i^—Mn1—N1—C1	36.82 (19)
C9—C10—C11—C6	0.4 (4)	O2W^i^—Mn1—N1—C1	−52.81 (19)
C12—C10—C11—C6	179.6 (2)	O2W—Mn1—N1—C1	127.19 (19)
C11—C10—C12—C13	9.7 (4)		

Symmetry codes: (i) −*x*+2, −*y*+1, −*z*−1; (ii) −*x*+3, −*y*+1, −*z*−1.

### Hydrogen-bond geometry (Å, °)

**Table:**

*D*—H···*A*	*D*—H	H···*A*	*D*···*A*	*D*—H···*A*
O3W—H3WA···O2^iii^	0.83 (2)	1.91 (2)	2.738 (2)	171 (3)
O1W—H1WA···O1^iv^	0.83 (2)	1.89 (2)	2.719 (2)	174 (3)
O2W—H2WA···O3W^v^	0.82 (2)	2.02 (2)	2.838 (3)	176 (3)
O3W—H3WB···O2^vi^	0.84 (2)	1.90 (2)	2.741 (2)	174 (3)
O2W—H2WB···O1^i^	0.83 (2)	1.88 (2)	2.702 (2)	171 (3)

Symmetry codes: (iii) −*x*+1, −*y*+1, −*z*; (iv) −*x*+2, *y*−1/2, −*z*−1/2; (v) −*x*+1, *y*−1/2, −*z*−1/2; (vi) *x*−1, *y*, *z*; (i) −*x*+2, −*y*+1, −*z*−1.
